# ﻿Two new tuberous species of *Begonia* (Begoniaceae) from Guangxi, China

**DOI:** 10.3897/phytokeys.256.145725

**Published:** 2025-05-21

**Authors:** Renchuan Hu, Jinye Zhou, Ziyi Zhao, Kedao Lai, Yannong Wu, Daike Tian

**Affiliations:** 1 Guangxi Key Laboratory of Traditional Chinese Medicine Quality Standards, Guangxi Institute of Chinese Medicine and Pharmaceutical Science, Nanning 530022, China Guangxi Institute of Chinese Medicine and Pharmaceutical Science Nanning China; 2 Flower Research Institute, Guangxi Academy of Agricultural Sciences, Nanning, Guangxi 530007, China Guangxi Academy of Agricultural Sciences Nanning China; 3 Chenshan Research Center of CAS Center for Excellence in Molecular Plant Sciences, Shanghai 201602, China Chenshan Research Center of CAS Center for Excellence in Molecular Plant Sciences Shanghai China; 4 Eastern China Conservation Centre for Wild Endangered Plant Resources, Shanghai Chenshan Botanical Garden, Shanghai 201602, China Shanghai Chenshan Botanical Garden Shanghai China

**Keywords:** *Begonia*, Begoniaceae, Guangxi, morphology, new taxon, South China

## Abstract

Two new tuberous species of *Begonia* (Begoniaceae), *Begoniaangustibracteata* D.K.Tian & R.C.Hu and *B.baishishanensis* R.C.Hu & D.K.Tian from Guangxi of China are described with illustrations. *Begoniaangustibracteata* has sparsely hairy green leaves, undivided placenta, and a small bracteole at the top of the pedicel of pistillate flower. *Begoniabaishishanensis* has sparsely hispidulous and unlobed leaves, bilaminate placenta and early flowering in March. The differences of these two new species are compared with their allied species, *B.fimbristipula* and *B.danxiaensis.* Their conservation status is assessed according to Guidelines for Using IUCN Red List Categories and Criteria.

## ﻿Introduction

*Begonia* L. is one of the largest genera of vascular plants, with 2,164 species worldwide (Hughes et al. 2015–). The habitat of this genus is mainly located under moist and shady forests (Clement et al. 2005). It is characterized by bright and beautiful flowers, diverse leaf shapes and colors, long flowering period, and easy cultivation, and therefore has long been developed as ornamental plants (Tian et al. 2018; Moonlight et al. 2023). There are many hybrids and cultivars available on the horticultural markets (Moonlight et al. 2018). China is rich in diversity of *Begonia*, with about 290 taxa (including subspecies and varieties) so far (Tian et al. 2024a, b, c; Wang et al. 2024; Zhou et al. 2024). Guangxi Zhuang Autonomous Region is located in southern China, on the southeastern edge of the Yunnan-Guizhou Plateau, and is the second step of China’s terrain. It is dotted with typical karst and Danxia landforms, which provide unique habitats for promoting species formation (Myers et al. 2000). A total of 262 families, 1,793 genera, and 8,793 species (including infraspecies) of wild vascular plants have been recorded in Guangxi, fully reflecting the richness of Guangxi’s plants (Wei et al. 2023).

In April 2020, during a field survey in Guiping City of Guangxi, we collected two tuberous taxa of *Begonia* that were quite different from the other known species. Through years of follow-up investigation, and comparing morphological characteristics with other allied species, we confirmed that these two taxa are new to science and named them as *B.angustibracteata* D.K.Tian & R.C.Hu and *B.baishishanensis* R.C.Hu & D.K.Tian, respectively. *Begoniaangustibracteata* belongs to BegoniasectionReichenheimia with tuberous habit and unilamellate placenta, and *B.baishishanensis* belongs to B.sectionDiploclininum with tuberous habit and bilamellate placenta. Their conservation status are assessed as Least Concern (LC) and Critically Endangered (CR), respectively, according to Guidelines for Using IUCN Red List Categories and Criteria (IUCN 2024).

## ﻿Material and methods

The specimens were collected from the evergreen broad-leaved forest of Guiping and Hengzhou City, and Danxia landform of Baishishan in July 2023, April 2024 and July 2024, respectively, and preserved in the herbaria of CSH and GXMI. Guiping City is located in the southeastern Guangxi of China, between latitudes 22°52′–23°48'N and longitudes 109°49′–110°22'E. It features a subtropical monsoon climate, with an annual average temperature of 22.4 °C, a relative humidity of 80%, abundant rainfall (average annual precipitation of 1726.7 mm), and ample sunshine. The topography of Guiping City is characterized by elevated terrain at the northern and southern ends, sloping toward a central plain. The vegetation within its boundaries is predominantly composed of *Pinusmassoniana* Lamb. (Masson pine) forests, interspersed with a small extent of subtropical evergreen broadleaf forests (Wei et al. 2024). Hengzhou City, also situated in southeastern Guangxi, lies to the east of Nanning City, with latitudes 22°08′–23°30'N and longitudes 108°48′–109°37'E. It exhibits a southern subtropical monsoon climate, characterized by intense solar radiation, abundant sunlight, warm temperatures, plentiful rainfall, long summers, short winters, an extended frost-free period, and rare occurrences of ice or snow. The terrain of Hengzhou City is encircled by mountains, with a gently expansive central area, steep northern ridges, southwestern hills, and isolated limestone peaks in the northeast, resembling a basin-like topography. The vegetation in Hengzhou City primarily consists of plantation forests, such as eucalyptus (*Eucalyptus* spp.) and Masson pine forests (Hengzhou Local Chronicles Compilation Committee Office 2024). The photos were taken with Nikon D750 and Nikon Z7. A detailed comparison was made with all other heretofore known *Begonia* species, including specimens deposited at the herbaria in China and descriptions from botanical websites (e.g. http://padme.rbge.org.uk/begonia/, http://www.cvh.ac.cn/, https://plants.jstor.org/). The conservation status of the new species is assessed according to Guidelines for Using IUCN Red List Categories and Criteria (IUCN 2024).

## ﻿Taxonomic treatment

### ﻿ Begonia angustibracteata

Taxon classificationPlantaeCucurbitalesBegoniaceae

D.K.Tian & R.C.Hu sp. nov.

7308B2AA-D05F-5D4E-BCF3-2BA656D4C2B9

urn:lsid:ipni.org:names:77362018-1

**Figure 1.**
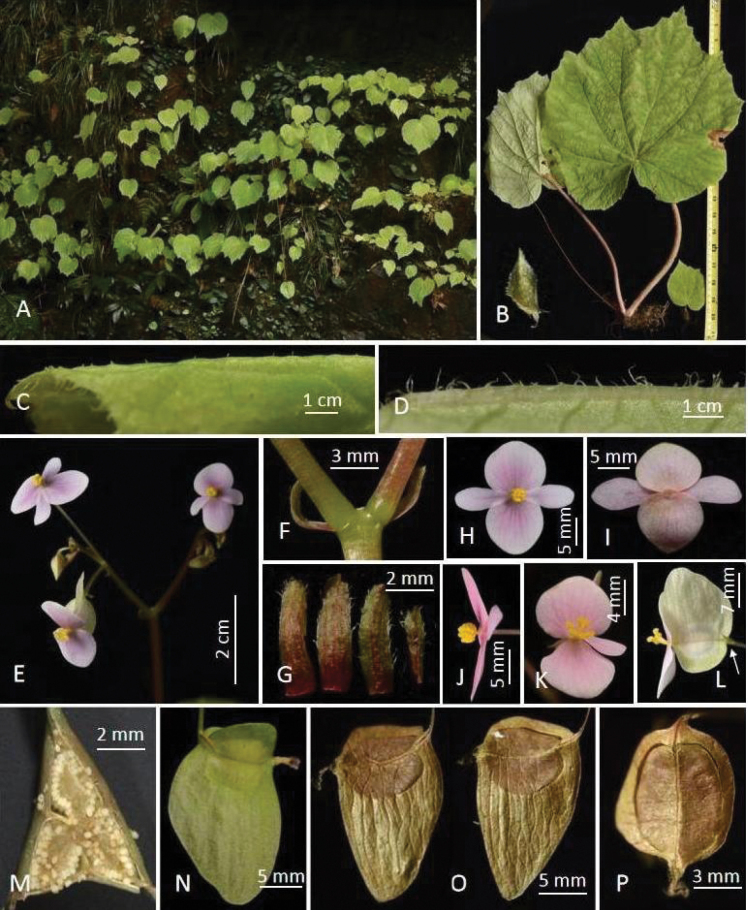
*Begoniaangustibracteata* D.K.Tian & R.C.Hu. **A** habitat and habit **B** large stipule (left corner) and small (lower right) individuals **C, D** portion of leaf, adaxial (**C**) and abaxial (**D**) surface **E** inflorescence showing flowers **F** peduncle showing basal bracts **G** bracts, abaxial views **H–J** front (**H**), back (**I**) and side (**J**) views of staminate flower **K** front view of pistillate flower **L** side view of ovary showing a bracteole at arrow **M** cross-section of ovary showing placentae **N** side view of fruit **O** side view of dried fruits showing dorsal wing **P** adaxial view of dried fruit. (Photos by Daike Tian)

Fig. 1 , Table 1 Chinese name: 狭苞秋海棠

#### Diagnosis.

The new species is mostly similar to the green-leaved individuals of *B.fimbristipula* Hance (Hance 1883), but differs mainly by its leaves sparsely hairy (vs. densely hairy), with a bracteole at the top of the pistillate flower pedicel (vs. without), larger (10–12 × 12–13 vs. 6–8 × 9–11 mm) outer tepals of pistillate flower, placentae unilamellate (vs. bilamellate), and later flowering (June–July vs. April–May).

#### Type.

**Figure 2.**
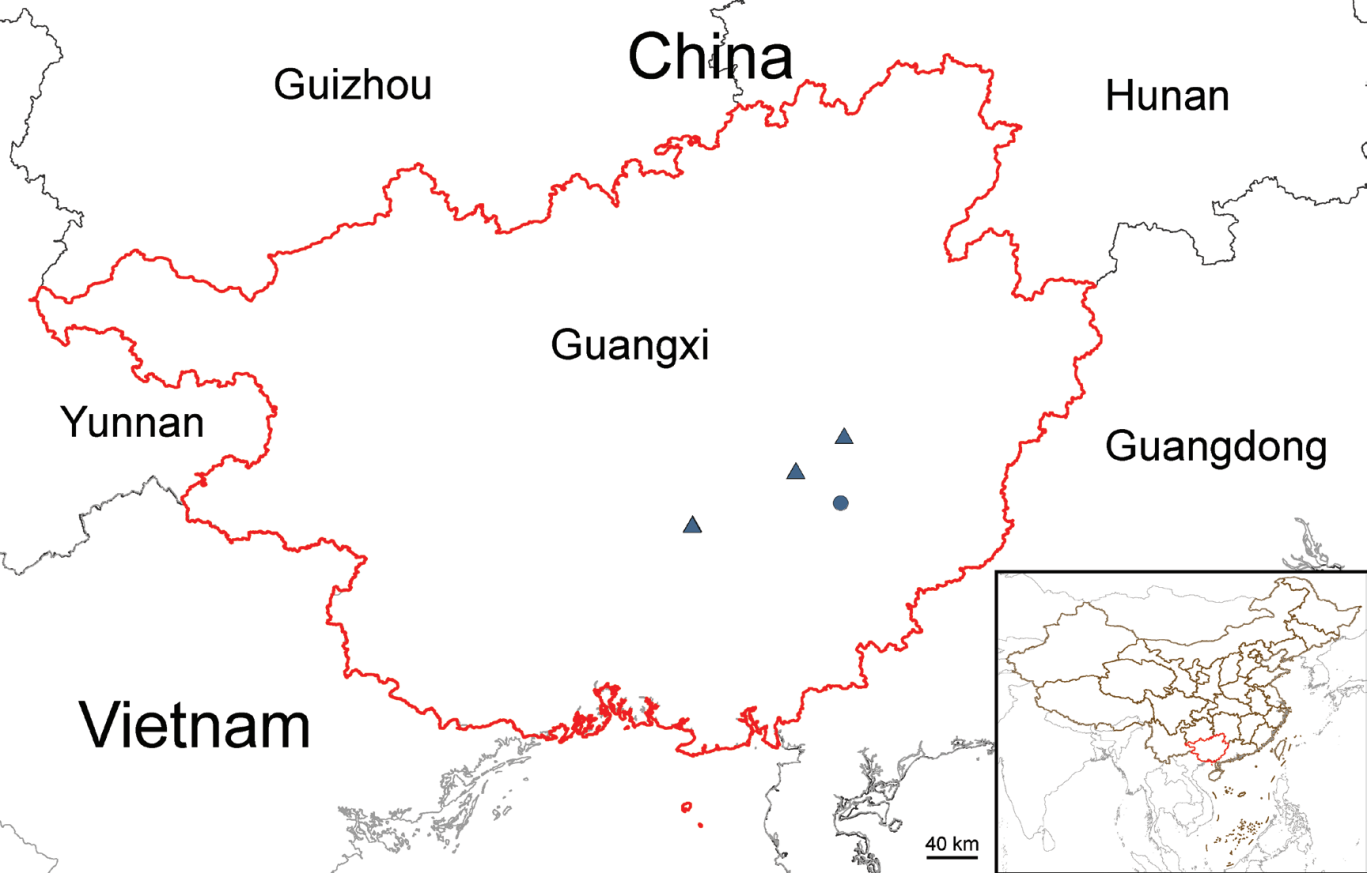
Distribution of *Begoniaangustibracteata* (black triangle) and *B.baishishanensis* (black dot) in Guangxi, China.

China. • Guangxi: Guiping (桂平) City, Xishan (西山) Town, Bitan (碧滩) Village (Fig. 2), near streams or on wet or slightly dry rock walls, 23°25'45"N, 109°53'9"E, elev. 38 m, dried fruits of the previous year, April 21, 2024, *Daike Tian*, *Jinye Zhou*, *Renchuan Hu* TDK5598 (holotype: CSH0214187; isotypes: CSH0214184, 0214185, 0214186, CSH!).

#### Description.

***Herb*** perennial, 2–20 cm tall, monoecious, tuber spherical, 8–25 mm in diameter. ***Leaves*** usually 1 per plant, sometimes 2 (more common in large plants); membranous, slightly asymmetric to near symmetrical, cordate, oval or ovate, 5–38 × 4–32 cm, wide side 2.2–17.5 cm, narrow side 2–15 cm, adaxially green, covered with very short rough hairs, hairs ≤ 0.5 mm, venation palmate, veins 10–11 (–12), concave; abaxially grayish-green, veins convex, covered with grayish-white villous, hairs 1–5 mm long, longer at the base of primary veins; margin heavily serrate and short grayish-green hairy, hairs ca. 1 mm long, apex acute and often coarsely toothed. ***Petioles*** green, light green or pink, 1.5–20 cm long, 1.5–9 mm thick, light green longitudinal spotted and grayish-white villous, hairs ≤ 6 mm long, or sparsely pale-white hairs on the upper of petioles; petiole base grooved. ***Inflorescence***: dichasial cyme, 1 per plant, occasionally 2, 5–33 cm long; peduncle 3–23 cm long, 2–6 mm thick, reddish-brown, glabrous, 3–10 flowers per inflorescence. ***Bracts*** nearly persistent, nearly linear, 3–6 × 1–2 mm, basal bracts with 3 red longitudinal stripes, upper bracts with red midribs. ***Staminate flower***: pedicel light green, usually gradually turning pink downwards, glabrous, 10–50 mm long, 1 mm thick; flower 16–23 × 18–25 mm; tepals 4, glabrous, outer 2 ovate, 8–12 × 8–12 mm, adaxially pink, slightly light green on the top edge, depressed and slightly thick in central part; abaxially color slightly lighter, nearly pinkish-white; inner 2 narrowly oblanceolate, pinkish-white, 9–12 × 4–5 mm, lighter and thinner than outer tepals; androecium nearly capitate, actinomorphic, 1.5–2 mm long, 2–3 mm wide; stamens 27–45, filaments basally fused, stamen column nearly 2 mm long, filaments and anthers nearly 1 mm long, fragrance pleasant. ***Pistillate flower***: pedicel light green, glabrous, 20–24 mm long, ca. 1 mm thick, with a bracteole on the top of pedicel or below ovary, 4–5 × ca. 1.5 mm; flower 13–21 × 12–14 mm, tepals 3, glabrous, outer 2 ovate, pinkish-white, slightly flat, the middle and lower parts gradually turning dark pink, 10–12 × 12–13 mm; inner 1 oblanceolate; pistils 4–5 × 3–4 mm, styles 3, nearly free, ca. 2 mm long; ovary 3 locular, placentation axile, placentae unilamellate. ***Fruit***: stalk 10–28 mm long, ca. 1 mm thick; capsule 6–9 × 4–6 mm, unequally 3-winged, large one nearly triangular or rectangular-triangular, 10–18 × 9–19 mm, lateral wings sickle-shaped, 1.5–3 × 8–10 mm.

**Table 1.** Morphological comparison of *Begoniaangustibracteata*, *B.fimbristipula*, *B.baishishanensis* and *B.danxiaensis*.

Characters	*B.angustibracteata*	*B.fimbristipula*	*B.baishishanensis*	*B.danxiaensis*
Leaf shape and texture	Cordate, oval or ovate, thin, unlobed to upper shallowly lobed	Broadly ovate, thin, unlobed	Cordate, thick, unlobed	Ovate-cordate to orbicular-reniform, very thin, shallowly lobed or crenate to biserrate
Blade size (cm)	5–38 × 4–32	4–13 × 4.8–8.5	3–21 × 2.6–20.3	2–13 × 1.5–13.5
Blade adaxial indumenta	Sparsely hispidulous, ≤ 0.5 mm long	Sparsely pubescent, ≥ 1 mm long	Sparsely hispidulous, ≤ 0.5 mm long	Sparsely hispidulous, ≤ 0.5 mm long
Bract	Margin hairy, 3–6 × 1–2 mm	Glabrous, 3–4 × 1.5–2.5 mm	Margin hairy, 4–12 × 1.5–5 mm	Glabrous, 4–12 × 1.5–3 mm
Outer tepals of staminate flower (mm)	8–12 × 8–12	8–13 × 8–10	13–17 × 10–14	4–11 × 3–10
Outer tepals of pistillate flower (mm)	10–12 × 12–13	6–8 × 9–11	10–13 × 11–15	3–9 × 4–9
Stamen number	27–45	Unknown	47–88	10–38
Pedicel of pistillate flower	With bracteole	Without bracteole	Without bracteole	Without bracteole
Placenta	Unilamellate	Bilamellate	Bilamellate	Unilamellate
Flowering time	June–July	April–May	March–April	April–May

#### Phenology.

Flowering June–July, fruiting July–August.

#### Etymology.

The epithet “*angustibracteata*” refers to the narrow bracts of this new species. The Chinese name is given as “狭苞秋海棠”.

#### Distribution and habitat.

The new species has been found only in three places restricted to Guiping City and Hengzhou City of Guangxi (Fig. 2). It grows on rock cliffs or in shaded places of valleys at an altitude of 30–300 m.

#### Conservation status.

The new species has been found only in three places of Guiping and Hengzhou City (Fig. 2), the extent of occurrence is about 210 km^2^ and the area of occupancy is about 12 km^2^. Although one of three locations has a large number of individuals (>10000 mature individuals, and many seedlings) and new populations will be possibly discovered in other places in the future. The conservation status of this species is assessed as Endangered (EN) (B1ab(i-v)+2ab(i-v)) due to possibly partial loss of habitats caused by road construction or maintenance, following Guidelines for Using the IUCN Red List Categories and Criteria (IUCN 2024).

#### Additional specimens examined.

China. • Guangxi: Guiping City, Xishan Town, Bitan Village, near streams on wet or slightly dry rock walls, or in crevices, 23°25'45"N, 109°53'9"E, elev. 38 m, flowering, July 26, 2023, *Renchuan Hu*, *Xincheng Qu 450881230726019* (GXMI!); • ibid., 23°25'58"N, 109°53'23"E, elev. 57 m, few dried fruits of the previous year, April 21, 2024, *Daike Tian*, *Jinye Zhou*, *Renchuan Hu TDK5599* (CSH!). • Guiping City, Jiangkou (江口) Town, Litang (理塘) Village, growing on rock wall or in crevices between rocks of Danxia landform, 23°40'57"N, 110°13'44"E, elev. 299 m, April 21, 2024, few dried fruits of the previous year, *Daike Tian*, *Jinye Zhou*, *Renchuan Hu TDK5601* (CSH!); • Hengzhou (横州) City, Zhenlong (镇龙) Town, growing on wet rock wall or in rock crevices, along a river valley in Jiulong Waterfall Group National Forest Park (九龙瀑布群国家森林公园), 23°3'23'N, 109°13'39"E, elev. 264 m, few dried fruits of the previous year, April 25, 2024, *Daike Tian*, *Jinye Zhou*, *TDK5639* (CSH!); • ibid., 23°3'0"N, 109°13'39"E, elev. 237 m, without flower, April 25, 2024, *Daike Tian*, *Jinye Zhou*, *TDK5642* (CSH!).

### ﻿ Begonia baishishanensis

Taxon classificationPlantaeCucurbitalesBegoniaceae

R.C.Hu & D.K.Tian sp. nov.

E5760856-20B5-5816-8EEB-5195C333F84C

urn:lsid:ipni.org:names:77362017-1

Fig. 3 , Table 1 Chinese name: 白石山秋海棠

#### Diagnosis.

*Begoniabaishishanensis* is most similar to *B.danxiaensis* D.K.Tian & X.L.Yu (Tian et al. 2019), but differs in its larger (3–21 × 2.6–20.3 cm vs. 2–13 × 1.5–13.5 cm) and thicker (vs. thinner) leaves, hairy (vs. nearly glabrous) bract margins, larger outer tepals of staminate flower (13–17 × 10–14 mm vs. 4–11 × 3–10 mm) and pistillate flower (10–13 × 11–15 mm vs. 3–9 × 4–9 mm), more (47–88 vs. 10–38) stamens, and placentae bilamellate (vs. unilamellate). The species is also similar to *B.fimbristipula* (Hance, 1883) belonging to B.sectionDiploclininum, but mainly differs by its larger (3–21 × 2.6–20.3 cm vs. 4–13 × 4.8–8.5 cm) and thicker (vs. thinner) leaves, shorter (≤ 0.5 mm vs. ≥ 1 mm long) hairs on adaxial leaf surface, larger outer tepals of pistillate flower (13–17 × 10–14 mm vs. 8–13 × 8–10 mm) and pistillate flower (10–13 × 11–15 mm vs. 6–8 × 9–11 mm).

**Figture 3.**
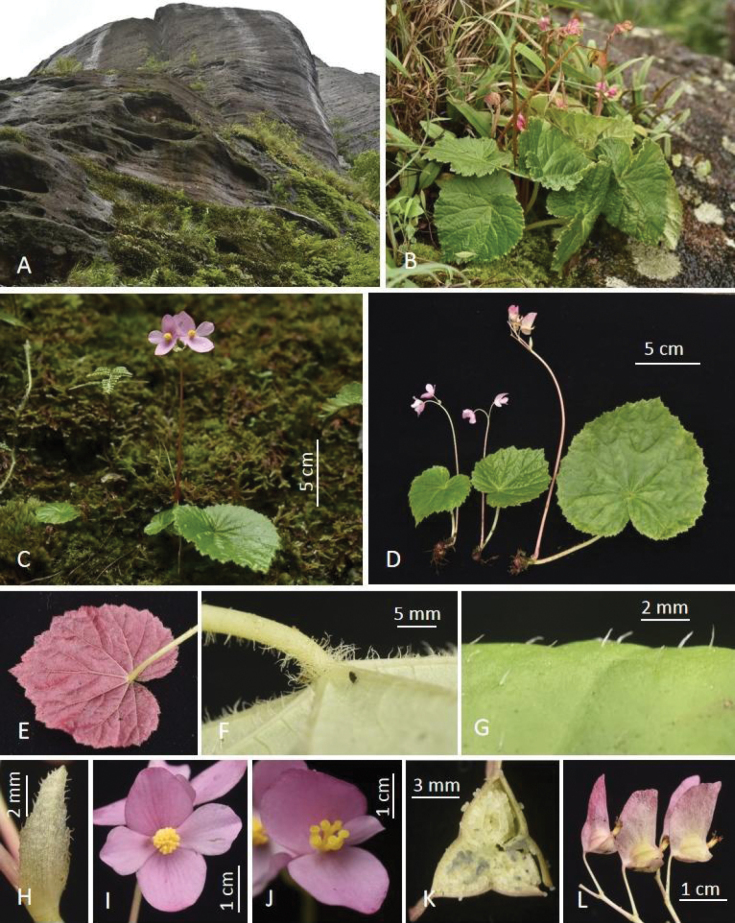
*Begoniabaishishanensis* R.C.Hu & D.K.Tian **A** habitata in Baishishan landform in Guiping of Guangxi **B, C** habit and plants in late flowering **D** individual plants showing size variation **E** abaxial blade surface of red leaf individual **F** abaxial leaf surface and petiole showing indumentum **G** adaxial leaf surface **H** bract **I** front view of staminate flower **J** front view of pistillate flower **K** cross-section of ovary showing bilamellate placentae **L** young fruits showing shape variation of large wings. (Photos by Daike Tian).

#### Type.

China. • Guangxi; Guiping City, Madong (麻垌) Town, Baishi (白石) Village (Fig. 2), growing on rock walls or with grasses of Danxia landform mountain, 23°42'8"N, 110°12'19"E, elev. 383 m, late flowering, April 21, 2024, *Daike Tian*, *Jinye Zhou*, *Renchuan Hu TDK5596* (holotype: CSH0214176; isotypes: CSH0214177, 0214178, 0214179, CSH!).

#### Description.

***Herb*** perennial, 3–15 cm tall, monoecious, tuber spherical, usually 2–3 connected, 8–30 mm in diameter; erect stem absent or with 1 at anthesis, 0.2–4 cm long, 3–8 mm thick. ***Stipules*** long triangular, ca. 6 mm long, covered with grayish-white long rough hairs. ***Leaves*** usually 1, rarely 2 per plant, blade cordate, nearly symmetrical, 3–21 × 2.6–20.3 cm, wide side 1.4–10.1 cm, narrow side 1.2–10.1 cm; adaxially green, rarely dark green, covered with grayish-white short rough hairs, hairs usually ≤ 1 mm long, venation palmate, veins 9–10, concave; abaxially gray-green, rarely purple-red, grayish-white villous, hairs 1–5 mm long, longer (up to 4 mm) at the base of primary veins, primary veins convex, secondary veins slightly convex; lobes of leaf base valvate to acute angled or slightly overlapped, margin with few serrate and short cilia, apex acuminate or short acuminate. ***Petioles*** green, rarely red, 0.8–18 cm long, 1.2–6 mm thick, grayish-white villous, hairs up to 4 mm long, denser on the top position. ***Inflorescence***: dichasial cyme, basal, usually 1, rarely 2 per plant, 4–40 cm long, peduncle 1.5–24 cm long, 0.8–8 mm thick, nearly glabrous, 2–15 flowers per inflorescence. ***Bracts*** light green, long triangular to nearly lanceolate, 4–11 × 1.5–5 mm, margin ciliate. ***Staminate flower***: pedicel greenish-white with pink, glabrous, 10–19 mm long, 0.6–0.8 mm thick; flower 26–34 × 25–31 mm, tepals 4, pink, coloration even, glabrous; outer 2 ovate or oval, 13–17 × 10–14 mm; inner 2 nearly obovate or obovate-lanceolate, 11–19 × 7–9 mm; androecium nearly capitate, actinomorphic, 3–4 × 4–5 mm; stamens 47–88; filaments base fused, connate part ca. 1.5 mm long, free part 1–1.5 mm long, anthers 0.8–1 mm long. ***Pistillate flower***: pedicel pink, glabrous, 8–13 mm long, 0.6–0.8 mm thick; flower 22–26 × 12–19 mm, tepals 3, pink, coloration nearly even, glabrous; outer 2 broadly ovate or ovate, slightly flat, 10–13 × 11–15 mm; inner 1 obovate or obovate-lanceolate, 6–12 × 3–7 mm; pistils 4–5 × 3–5 mm; styles 3, nearly free, 1.5–2 mm long; ovary 3 locular, placentation axile, placentae bilamellate. ***Fruit***: stalk 11–20 mm long, ca. 1 mm thick, capsule 8–11 × 6–9 mm, unequally 3-winged, larger one nearly rectangular or triangular-rectangular, 11–15 × 10–15 mm, apex nearly obtuse or pointed; smaller wings sickle-shaped, 1–2 × 7–16 mm.

#### Phenology.

Flowering March to April, fruiting April to May.

#### Etymology.

The epithet “*baishishanensis*” refers to Baishishan, a mountain name of Guiping City and the type locality of this species. The Chinese name is given as “白石山秋海棠”.

#### Distribution and habitat.

One population including four subpopulations of *B.baishishanensis* are found on rock walls or grass, or in rock crevices of Danxia landform cliff of Baishishan of Guiping City, Guangxi, China (Fig. 2).

#### Conservation status.

The new species is only found in the type locality, the extent of occurrence is about 2 km^2^ and the area of occupancy is less than 2 km^2^, and has only one population (< 2000 mature individuals, and few seedlings). Due to its unique habitat and negative influence by tourism, its living space is shrinking, currently it is assessed as Critically Endangered (CR) (B1ab(i–v)+2ab(i–v)) following Guidelines for Using the IUCN Red List Categories and Criteria (IUCN 2024). Therefore, *B.baishishanensis* should be suggested to be included in the future List of China National Protected Wild Plants. However, further investigation is necessary on its actual distribution area and the number of populations and individuals.

#### Additional specimens examined.

China. • Guangxi: Guiping City, Madong Town, Baishi Village, growing on rock wall or grass of Danxia landform mountain, 23°42'8"N, 110°12'19"E, elev. 383 m, peak flowering, March 25, 2024, *Renchuan Hu Xincheng Qu*, *TDK5596* (CSH!).

## ﻿Discussion

These two new species are distributed in Guiping City, where the terrain is high in the north and south, low in the central plain, forming a natural barrier and a typical subtropical monsoon climate. In addition, with abundant water resources and superior natural conditions, the city is characterized by rich plant resources and high species diversity. It is worth noting that this climate zone is one of the regions with the highest distribution of *Begonia*, such as *B.shenzhenensis* D.K.Tian & X.Yun Wang (Tian et al. 2021), *B.danxiaensis* (Tian et al. 2019), *B.huangii* Y.M.Shui & W.H.Chen (2005) and *B.longistyla* Y.M.Shui & W.H.Chen (2005), etc. In addition, many plants of *Begonia* have also been discovered in the same climate zone of Brazil (Jacques et al. 2018; Jaramillo et al. 2018; Funez et al. 2019). In recent years, scientists have discovered and recorded an increasing number of new plant species in this climate zone, including Meliaceae (Nong et al. 2023), Pteridaceae (Chu et al. 2021), and Loganiaceae (Hu et al. 2000), etc. As biodiversity surveys progress further, it is expected that more new species will be discovered and published in this climate zone around the world especially in China (Chen et al. 2019).

## Supplementary Material

XML Treatment for Begonia angustibracteata

XML Treatment for Begonia baishishanensis

## ﻿Additional information

### Author ORCIDs

Renchuan Hu https://orcid.org/0000-0002-0941-7203

Jinye Zhou https://orcid.org/0000-0003-2357-3437

Ziyi Zhao https://orcid.org/0000-0003-2513-0728

Daike Tian https://orcid.org/0000-0001-5387-5300

### Data availability

All of the data that support the findings of this study are available in the main text.
